# Genito-Urinary and Syphilitic Diseases

**Published:** 1899-08

**Authors:** Byron H. Daggett

**Affiliations:** Buffalo, N. Y.


### GENITO-URINARY AND SYPHILITIC DISEASES.

CONDUCTED BY BYRON H. DAGGETT, M. D., Buffalo, N. Y.

### SOME OF THE CLINICAL ASPECTS OF GRANULAR KIDNEY.

WEST, SAMUEL, before the London Medical Association stated that granular kidney is a disease of more importance and exists more frequently than is generally supposed, since it is frequently unrecognised. It is often unsuspected until demonstrated by post mortem. It is found after death in other diseases which might not have resulted fatally but for the presence of granular kidney. It is often overlooked; it is often unexpectedly found; it often supplies the missing link in obscure cases. Thus it is one of the most interesting as well as one of the most important pathologic conditions. Post mortem revealed granular kidney in a large percentage of 79 cases of autopsies—excluding children under five years of age—brought to St. Bartholomew's Hospital, either dead or dying; 48 per cent. had chronic interstitial nephritis, which was the only cause of death in 16.8 per cent. This malady and its resulting lesions caused death in 21.6 per cent more, that is, it was the sole or contributing cause of death in 38.4 per cent. of these cases.

Ten per cent. of 100 fatal cases of acute pneumonia showed, upon post mortem, granular kidney. Granular kidney is a bilateral and to a great extent a symmetrical affection. The kidneys are generally reduced in size and weight, occasionally they are larger and heavier than normal. There are large as well as small granular kidneys, just as there are large and small cirrhotic livers. The granular condition of the interstitial tissue is readily noted by microscopical examination.

The typical granular kidney is small, contracted, hard, cirrhotic, nodular and the surface is often well covered with cysts. Section of the kidney shows cortical substance, infiltrated with fibroid indurations and cellular degeneration. Regarding the question of inflammation, there is no more evidence of this process in the cirrhotic kidney than there is in the cirrhotic liver. The contracted kidney is divided into two forms, the red and the white. They are not microscopically distinguishable. Clinically they are not distinguishable; pathologically the question of distinction is unsettled.

Chronic interstitial nephritis is often described as granular kidney, but chronic interstitial nephritis is not necessarily granular. For instance, unilateral lesion, due to obstructed ureter, may not be granular. Fatty degeneration, fibrosis, infarcts and grumata, even if invading the parenchyma, are excluded, they do not produce the clinical symptoms of granular kidney. Degenerate kidneys due to atheroma, gout and age are not classified as granular.

Granular kidney is divided into arterial, sclerotic and renal, and are not clinically distinguishable. Whether the primary cause of the disease is in the vessels or the kidney itself is an important and an issue not yet settled. The symptoms of acute parenchymatous nephritis are well marked, while those of granular kidney are insidious and unrecognised. There is no relationship shown, either clinically or in any other way twixt acute nephritis and granular kidney. Attacks of parenchymatous may occur through a series of years, without albuminuria; without causing thickening of the arterio or cardiac hypertrophy and without causing retinæ change. In fact without producing clinical signs of granular kidney. Acute nephritis may result in granular kidney, but granular kidney does not cause acute nephritis. Granular kidney may precede acute parenchymatous nephritis. There are recognised forms of pathologic arteries—atheroma and thickening of the vascular walls, due to muscular hypertrophy and changes that are not atheromatous. Whether this condition is primarily arterial or renal is a mooted question; at all events, granular kidney is a disease *sui generis* and should be treated accordingly. Granular kidney is a very insidious disease, its symptoms only occur when the disease is well advanced. The first symptom is frequent micturition at night, and this is not constant. The symptoms are cardio-vascular and renal. The cardiac symptoms are those of cardiac failure, varying in degree. The vascular symptoms are hemorrhagic and arterial. The defective action of the kidneys causes renal toxemia.

The signs and symptoms are : (1) physical signs; (2) the cardio-vascular symptoms; (3) the renal symptoms.

The early signs of the disease are the most important. In the early stage the diagnosis is to be made by physical signs, which are high tension pulse, thickened arteries, hypertrophy of the heart and albuminuria. Hypertrophy of the heart is probably secondary to the vascular changes and the result of them. Thickening of the arteries is a cardinal sign of this disease, and is never absent in advanced cases. Albuminuric retinitis is an early sign. High tension pulse and thickened arteries, in the young, indicate the initial stage of granular kidney.

